# Epigenetic regulation of bone remodeling and its role in the pathogenesis of primary osteoporosis

**DOI:** 10.18699/VJGB-23-48

**Published:** 2023-07

**Authors:** B.I. Yalaev, R.I. Khusainova

**Affiliations:** Institute of Biochemistry and Genetics – Subdivision of the Ufa Federal Research Center of the Russian Academy of Sciences, Ufa, Russia Saint Petersburg State University, St. Petersburg, Russia; Institute of Biochemistry and Genetics – Subdivision of the Ufa Federal Research Center of the Russian Academy of Sciences, Ufa, Russia Saint Petersburg State University, St. Petersburg, Russia Ufa University of Science and Technology, Ufa, Russia

**Keywords:** osteoporosis, methylation, microRNA, acetylation, остеопороз, метилирование, микроРНК, ацетилирование

## Abstract

Discovery of molecular mechanisms of primary osteoporosis development is fundamental to understand the pathogenesis of musculoskeletal diseases in general and for identifying key links in the genetic and epigenetic regulation of bone remodelling genes. The number of identified molecular genetic markers for osteoporosis is increasing but there is a need to describe their functional interactions. These interactions have been determined to be associated with the control of expression of a number of transcription factors and the differentiation of mesenchymal stem cells through the pathway of osteoblastogenesis or adipogenesis, and monocytic precursors through the pathway of osteoclastogenesis. The results of epigenetic studies have significantly increased the understanding of the role of post-translational modifications of histones, DNA methylation and RNA interference in the osteoporosis pathogenesis and in bone remodelling. However, the knowledge should be systematised and generalised according to the results of research on the role of epigenetic modifiers in the development of osteoporosis, and the influence of each epigenetic mechanism on the individual links of bone remodelling during ontogenesis of humans in general, including the elderly, should be described. Understanding which mechanisms and systems are involved in the development of this nosology is of interest for the development of targeted therapies, as the possibility of using microRNAs to regulate genes is now being considered. Systematisation of these data is important to investigate the differences in epigenetic marker arrays by race and ethnicity. The review article analyses references to relevant reviews and original articles, classifies information on current advances in the study of epigenetic mechanisms in osteoporosis and reviews the results of studies of epigenetic mechanisms on individual links of bone remodelling.

## Abstract (ru)

Раскрытие молекулярных механизмов развития первичного остеопороза имеет фундаментальное значение как с точки зрения понимания патогенеза заболеваний опорно-двигательного аппарата в целом, так и для выявления ключевых звеньев генетической и эпигенетической регуляции экспрессии генов ремоделирования костной ткани. Количество обнаруженных молекулярно-генетических маркеров остеопороза продолжает расти, однако существует очевидная необходимость описания их функциональных взаимодействий. Установлено, что такие взаимодействия сопряжены с контролем экспрессии ряда факторов транскрипции и дифференцировки мезенхимальных стволовых клеток по пути остеобластогенеза и адипогенеза, а моноцитарных предшественников – по пути остеокластогенеза. Кроме того, результаты эпигенетических исследований значительно расширили понимание роли посттрансляционных модификаций гистонов, ДНК-метилирования и РНК-интерференции как в молекулярном патогенезе первичного остеопороза, так и в регуляции развития костной ткани. Несмотря на это, знания не систематизированы и нуждаются в обобщении данных исследований роли эпигенетических модификаторов в развитии первичного остеопороза, и, что не менее важно, в описании влияния каждого известного эпигенетического механизма на отдельные молекулярные звенья процесса формирования и резорбции костной ткани в течение онтогенеза человека, в том числе у лиц пожилого возраста. Понимание того, какие молекулярно-генетические механизмы и регуляторные системы вовлечены в развитие данной нозологии, представляет потенциальный интерес для создания таргетной терапии, поскольку уже сейчас рассматривается вопрос о возможности применения микроРНК для узконаправленной регуляции генов. Кроме того, систематизация этих данных важна для изучения разницы массивов эпигенетических маркеров, в зависимости от расовой и этнической принадлежности. В представленной обзорной статье проанализированы соответствующие систематические обзоры и оригинальные статьи, собрана и классифицирована информация о современных достижениях в области изучения эпигенетических механизмов и их аберраций при первичном остеопорозе, а также рассмотрены результаты исследований эпигенетических механизмов на отдельных функциональных звеньях ремоделирования костной ткани.

## Introduction

Primary osteoporosis (OP) is an age-associated disease of multifactorial aetiology, which is based on a violation of the balance of bone remodelling, leading to a decrease in the level of bone mineral density (BMD) and a violation of the structure of bone microarchitectonics, which result in the appearance of an incorrect spatial structure of the spongy and cortical bone (Yalaev et al., 2021). Bone mass is reduced to 26 % in individuals predisposed to OP. In roughly 80 % of women, the mineral content in the spine falls below the threshold value at the age of 60–70 years, and at 85 years – in more than 90 % (Sveshnikov, 2013). According to statistics, the frequency of fractures in women is 33 %, in men, 20 % (Marshall et al., 1996; Estrada et al., 2012; Lee et al., 2020; Widl et al., 2020).

Until recently, OP was diagnosed only by secondary signs, such as low height and bone pain (Lorentzon, Cummings, 2015). In 1940, the American endocrinologist F. Albright, describing postmenopausal osteoporosis, suggested that it developed due to estrogen deficiency (Albright et al., 1940). On this basis, clinicians developed the now obsolete concept of two forms of OP, one associated with estrogen deficiency during menopause and the other with calcium deficiency and skeletal ageing, both of which are characteristic of both sexes (Riggs et al., 1982).

Current evidence defines OP as a musculoskeletal disease associated with profound metabolic changes not only in bone, but also in whole-body homeostasis: micro-nutrient and endocrine dysregulation, as well as a complex interaction of genetic, endogenous and environmental factors that contribute to a complex disease phenotype (Foger-Samwald et al., 2020). Risk factors with regards to OP are assumed to be the female sex, being of the Caucasoid or Mongoloid race (Thomas, 2007), early menopause, old age, family history, insufficient insolation, comorbidities with impaired bone matrix micronutrient absorption, smoking, alcohol abuse, sedentary lifestyle, taking hormonal drugs and rheumatoid arthritis. DNA testing has not been introduced into diagnostic practice because significant population differences in the frequency distribution of risk markers prevent the development of universal test systems, despite the existence of significant and validated genetic markers for OP (de Souza, 2010; Bolland et al., 2011).

The first multicentre molecular genetic studies based on the genome-wide association search (GWAS) method confirmed that OP is associated with genes for local and systemic regulation of bone cell function. The lion’s share of risk markers, specifically, single-nucleotide polymorphisms, have been identified not in exons but in introns and promoters of regulatory genes, transcription factor, receptor (ESR2) and growth factor (FGF2) genes (Rivadeneira et al., 2009; Estrada et al., 2012; Wood et al., 2015). Certain markers have been identified in non-coding RNA (ncRNA) sequences, including microRNA (Lei et al., 2011; Yalaev, Khusainova, 2020). Significant levels of associations with fractures have been identified among the genes of systemic and local regulators (e. g., RANKL and OPG), transmembrane receptors, as well as WNT signalling genes, nuclear transcription factors (ZNF239, etc.,) and enzymes that produce or inactivate local bone regulators (Raisz, 2005).

Bioinformatics studies have concluded that the main enrichment pathways by means of the functional affiliation of genes associated with histone modifications and microRNA patterns are significantly associated with the regulation of RUNX2, FGF2 and SOX9 transcription factors. Moreover, they are also associated with the regulation of mesenchymal stem cell (MSC) differentiation (Letarouilly et al., 2019). Thus, particular epigenetic factors in the pathogenesis of osteoporosis have been identified. However, at present, it is more interesting to consider the molecular pathogenesis of OP in terms of individual functional links in the regulation of bone remodelling, in particular how RANK/RANKL/ OPG, WNT, RUNX2 transcription factor and others are epigenetically regulated.

## The role of DNA methylation and microRNA in the regulation of the RANK/RANKL/OPG system in primary osteoporosis

Controlled differentiation of osteoblast and osteoclast precursors (mononuclear phagocytes) is necessary to maintain the balance of bone remodelling (Soltanoff et al., 2009). Osteoclast activity is primarily regulated by the RANK/ RANKL/OPG (receptor-activator nuclear factor kβ/RANK ligand/osteoprotegerin) system. RANKL is produced by osteoblasts and its binding to RANK on the osteoclast surface activates the expression of osteoclastogenic genes (Tobeiha et al., 2020).

The role of gene polymorphisms of this system in increased risk of fracture and the formation of low BMD has previously been shown (Yalaev, Khusainova, 2020). In the RANKL gene sequence, two CpG sites were detected: one in the upstream sequence with 18 CpG sites, localized at a distance of 14,415 pairs of nucleotides (bp) from the transcription start site of the TSS I major isoform and one in the downstream sequence with 59 CpG sites, which starts at 260 and ends at 615 pairs of nucleotides of the TSS I isoform. In the OPG gene sequence, one island of 56 sites spanning from –402 to +850 nucleotide pairs from the transcription site of the TSS isoform was observed (Delgado-Calle et al., 2012). In (Wang et al., 2018), a group of patients from Guangzhou Medical University Hospital with osteoporotic fractures had significantly higher RANKL gene mRNA levels from femoral bone cells compared to controls, with downregulated methylation status (Wang et al., 2018).

Dysregulation of the expression of these genes is known to be a key link in the development of steroid-induced osteonecrosis of the femoral head and was noted to be associated with increased DNA methylation levels of OPG and RANK genes and with decreased levels in the RANKL gene (Sun et al., 2021). Previously, J. Delgado-Calle et al. (2012) performed a comparative analysis of the expression levels and methylation profile of the RANKL and OPG genes in bone tissue samples from patients with osteoporotic femoral neck fractures and osteoarthritis (OA) of the hip joint, which obtained remarkable results. RANKL expression levels were significantly higher in patients with fractures ( p = 0.012), while no significant changes in OPG gene expression levels were observed. The ratio of RANKL ligand to OPG was higher in bone samples with OP (7.66 ± 0.23 versus 0.92 ± 0.21, p = 0.002). Differential methylation analysis revealed that the upstream promoter region of the RANKL gene was strongly methylated in all samples, while individual CpG junctions of the gene were equally hypomethylated in the comparison groups (Delgado-Calle et al., 2012).

There is evidence of the contribution of miRNA to the regulation of the RANK/RANKL/OPG system. In 2014, C. Chen et al. published results measuring miR-503 microRNA levels in peripheral blood cells, the overexpression of which in CD14+ mononuclear cells inhibited RANKL-induced osteoclastogenesis. In CD14+ cells from postmenopausal women with osteoporosis, the baseline level of miR-503 was lower than in normal controls and had no change after induction by RANKL factor, suggesting the direct role of miR-503 in the regulation of RANK expression (Chen et al., 2014). miR-142-3p and miR-21- 5p are potential biomarkers of OP as they have a high affinity with the OPG gene mRNA and are involved in the regulation of several signalling pathways involved in bone formation (Ge et al., 2007; Hu et al., 2020). Thus, the epigenetic regulation of the RANK/RANKL/OPG system is dynamic and dependent on the DNA methylation status and a number of microRNAs.

## The role of epigenetic mechanisms in the regulation of bone cell differentiation through alteration of RUNX2 activity

Transcription factor 2 (RUNX2) plays a crucial role in osteoblast differentiation. Gene expression is predominantly high in the early stages of bone cell development when MSCs are differentiating into osteoblast precursors, but naturally decreases at the osteocyte maturation stage (Stein et al., 2004).

The gene contains several functional regions: activation domain, runt domain, PST domain, etc. (Gomathi et al., 2020). In terms of epigenetic regulators of RUNX2, microRNAs are well studied. In particular, miRNA-194 modulates MSC differentiation (Gomathi et al., 2020) and accelerates osteoblast differentiation by regulating RUNX2 nuclear translocation by way of STAT1 signal transducer (Li J. et al., 2015). miR-133a-5p inhibits RUNX2 gene expression at the transcription and translation level by binding to the 3′-untranslated mRNA site (Zhang et al., 2018).

During the early stages of osteoblast maturation, miR- 125b affects RUNX2 expression by affinity binding to the 3′-untranslated region of the gene, indirectly participating in the formation of a complex with Cbfβ that inhibits differentiation of these cells. Using microarray technology, P. Garmilla-Ezquerra et al. (2015) discovered a significant reduction in miR-187-3p gene expression levels and induction of miR-518f in bone with low BMD. In their research, Y. Zhang et al. (2017) observed the involvement of miR-221 in the formation of low mineral density through regulation of RUNX2 activity based on bioinformatics analysis (Garmilla-Ezquerra et al., 2015; Zhang et al., 2017). Several microRNAs affecting the activity of this factor are presented in Table 1.

**Table 1.**
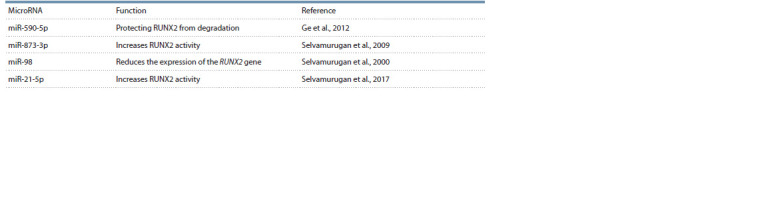
Factors involved in the regulation of RUNX2 expression

Phosphorylation of RUNX2 mobilises chromatin regulatory factors and accelerates MSC maturation. RUNX2 is phosphorylated at specific serine residues 301 and 319, inducing osteocyte maturation through MAPK-dependent signalling (Ge et al., 2009; Li Y. et al., 2017) and BMP2- sensitive transcription (Afzal et al., 2005). Phosphorylation of S104 leads to prevention of RUNX protein degradation (Huang et al., 2001; Wee et al., 2002). The ERK-MAPK signalling pathway plays a crucial role in the regulation of RUNX2 gene expression and bone formation (Ge et al., 2007). MKK6 is a protein kinase with dual specificity that participates in the MAP kinase signal transduction pathway and promotes RUNX2 phosphorylation (Ge et al., 2012). In addition, parathormone, which is one of the main regulators of blood calcium levels, activates the phosphorylation of the RUNX2 factor by means of the PKA signalling pathway. This process is associated with the activation of the promoter of the MMP13 gene, which plays an essential role in bone resorption (Selvamurugan et al., 2000, 2009).

Post-translational modification of histones, in particular histone methylation, plays an important role in bone formation. The so-called JUMONJI protein is considered to be a transcription factor and is encoded by the JARID2 gene. The domain containing 3 JMJD3 is a histone demethylase specifically catalysing the removal of trimethylation of histone H3K27me3. JMJD3 was found to inhibit the differentiation of RUNX2 osteoblasts. Conversely, inhibition of JMJD3 activity decreases RUNX2 promoter activity while increasing H3K27me3 activity in promoter regions (Yang et al., 2013).

Histone acetylation affects the state of chromatin compaction by neutralising the positive charge of histone tails and reducing the electrostatic interactions of histone tails with deoxyribonucleic acid. Studies have revealed that osteoporosis induced by glucocorticoid therapy leads to decreased acetylation of H3K9/K14 and H4K12 in the regulatory regions of the RUNX2 and OSX genes and increases the hyperacetylation of H3K9/K14 and H4K12 in the PPARγ2 regulatory region in bone marrow-derived MSCs from osteoporosis. The transcriptional activity of the RUNX2 factor gene is enhanced by P300 acetyltransferase and nicotinamide phosphoribosyltransferase (NAMPT), which, in turn, promote osteogenic differentiation of MSCs by H3K14 acylation and MC3T3-E1 cells through H3K9 acylation, respectively (Xu et al., 2021).

Thus, an extremely significant number of regulators of RUNX2 gene expression have been identified, which is a promising therapeutic gene model in culture studies, as blocking or enhancing the activity of this gene and monitoring its expression level during the induction of osteogenic lines allows the identification of key switches of mesenchymal stem cell differentiation.

## The role of epigenetic regulation of the WNT-signalling pathway in the regulation of bone remodelling and the pathogenesis of osteoporosis

The WNT signalling pathway is one of the most important systems regulating embryonic development and cell differentiation. This pathway represents one of the central links in the control of bone development and remodelling. Among the various genes involved in this system, methylation of the SOST (encoding sclerostin) gene promoter has been comprehensively studied in osteoblast cultures. Sclerostin produced by osteocytes inhibits WNT signalling and reduces the rate of bone formation. DNA methylation of the gene is necessary for the transition of osteoblasts to osteocytes (Delgado-Calle et al., 2012). In women with primary OP, SOST methylation is elevated in iliac bone cells whilst sclerostin levels are decreased and the WNT pathway is enhanced (Reppe et al., 2015).

Several studies have shown that histone deacetylation under the regulation of the WNT6, WNT10B, WNT10A and WNT1 genes inhibits WNT signalling and increases the risk of primary osteoporosis (Jing et al., 2018). Thus, elevated levels of HDAC5 deacetylase reduce SOST gene expression in osteocytes, contributing to bone mass loss. Deficiency of this deacetylase is associated with the acetylation of histone H3 lysine 27 as well as the interaction of MEF2C with the SOST gene enhancer, therefore suggesting the significant role of sclerostin in the regulation of osteocyte maturation (Wein et al., 2016). High levels of the zeste 2 homologue enhancer methyltransferase (EZH2) have been demonstrated to suppress osteogenic differentiation of MSCs, while low ones decrease the levels of the H3K27me3 tag near the transcription start site of osteogenesis genes, including WNT10B (Dudakovic et al., 2016). EZH2 increases H3K27me3 levels at the WNT1, WNT6 and WNT10A promoters and inhibits WNT signalling (Jing et al., 2016).

Many regulatory microRNAs are associated with WNT signalling. miR-433-3p inhibits DKK1 (Dickkopf-1) gene expression, enhancing osteoblast differentiation. Dickkopf-1 acts as an antagonist of the WNT signalling pathway and enhances bone resorption (Tang et al., 2017). miR-139-5p induces WNT signalling via the inhibition of NOTCH1 (Feng et al., 2020). R.E. Makitie et al. (2018) screened a specially designed panel of 192 microRNAs in patients with a genetically determined WNT signalling disorder with a heterozygous missense mutation c. 652 T>G (p. C218G) in exon 4 of the WNT1 gene. It was determined that miR-22-3p, miR-34a-5p and miR-31-5p levels were lower in mutation carriers compared to controls (Makitie et al., 2018).

Another common inhibitor of osteogenic differentiation is miR-31, the level of which drops in MSCs differentiating into osteoblasts. This was confirmed by S. Weilner et al. (2016), who observed increased levels of this microRNA in plasma in elderly patients with OP (Weilner et al., 2016; Amjadi-Moheb, Akhavan-Niaki, 2019). miR-31 is released from extracellular vesicles of endothelial cells and inhibits osteogenesis in stromal stem cells by binding to the Freisled 3 protein. Furthermore, decreased levels of miR-199a-5p result in glucocorticoid-mediated inhibition of osteogenesis (Shi et al., 2015). More recently, L. Duan et al. (2018) identified that high levels of miR-16-2* may contribute to the development of primary OP: knockdown of this microRNA may promote RUNX2 activation. This microRNA has an affinity for the WNT5A gene mRNA (Duan et al., 2018). miR-148a-3p has been revealed to enhance both osteoclastogenesis (Cheng et al., 2013) and adipogenesis in osteogenic progenitor cells (Gao et al., 2011). Plasma levels of this microRNA are significantly higher in patients with OP compared to controls without OP (Bedene et al., 2016). miR-30e is another important microRNA in the pathogenesis of OP, playing a role in regulating adipocyte and osteoblast differentiation through the inhibition of LRP6 (Wang et al., 2013). Thus, the WNT signalling pathway is regulated by a complex epigenetic system, notably microRNAs.

The role of non-coding RNAs in bone remodelling MicroRNAs are considered to be the most studied epigenetic factors in osteoporosis (Yalaev, Khusainova, 2020). They are conventionally divided into two classes: those that promote bone formation or bone resorption. In particular, several microRNAs that slow down the progression of OP have been identified. For example, miR-33-5p is a mechanosensitive microRNA that positively regulates osteoblastogenesis by way of inhibition of HMGA2 high mobility group proteins (Wang et al., 2016). miR-96 enhances osteogenic differentiation by inhibiting phosphorylation of epidermal growth factor receptor EGFR and the expression of major osteoblast factors RUNX2 and OSTERIX (Yang et al., 2014). miR-216a enhances bone formation by regulating the c-Cbl-mediated PI3K/AKT pathway (Li H. et al., 2015). Table 2 shows the microRNAs separated by direction of action in bone remodelling (Yalaev, Khusainova, 2018).

**Table 2.**
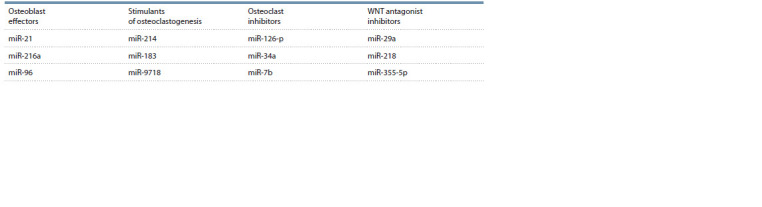
MicroRNAs stimulating bone formation or resorption

miR-124 is a positive regulator of adipogenic and neurogenic differentiation, while being a negative regulator of myogenic and osteogenic differentiation. It directly targets the DLX3, DLX5 and DLX2 homeobox genes (Qadir et al., 2015). In one pharmacogenetic study, patients with OP, after three months of treatment with the parathormone analogue Teriparatide, had reduced expression levels of miR-33 and one year later, miR-133a. Simultaneously, there was a general tendency: the increase in the level of BMD increased with a decrease in the level of expression of these microRNAs. Post-transcriptional regulation of DKK-1 changes due to a decrease in miR-33 microRNA levels and the action of parathyroid hormone, which leads to a decrease in the negative impact of DKK-1 on an alternative regulatory mechanism that improves optimal control of WNT signaling.(Anastasilakis et al., 2018).

Of interest are the results of studies on the effects of long non-coding RNAs on the regulation of Sirtuins, nicotinamide adenine dinucleotide-dependent deacetylases. These molecules have a broad spectrum of action and are associated with longevity and resistance to age-related diseases. It was established that SIRT1 gene expression is inversely related to HIF1A-AS1 ncRNA expression, while HOXA-AS3 ncRNA interacts with EZH2 and is required for RUNX2 trimethylation of lysine-27 H3 (H3K27me3) factor. Hence, HOXA-AS3 is important for bone formation in general (Yang et al., 2020).

The long ncRNA HOTAIR reduces protein expression and inhibits WNT signalling. DKK1 reduces the protein levels of C-myc, β-catenin, HOTAIR and RUNX2, which theoretically counteracts the regulatory effect of HOTAIR (Zhang et al., 2019). If the expression level of p21 ncRNA is low, WNT signalling becomes more active due to increased E2 secretion, which ultimately increases the rate of bone formation (Yang et al., 2019). Also, reduced levels of H19 ncRNA reduce the level of DKK4 gene expression (Li B. et al., 2017). AK045490 ncRNA levels are significantly elevated and inhibit bone formation by inhibiting nuclear translocation of β-catenin and suppressing TCF1, LEF1 and RUNX2 expression (Li et al., 2019). Similarly, AK016739 ncRNA inhibits osteogenic differentiation as it can reduce the expression and activity of osteoblastogenesis transcription factors (Yin et al., 2019).

Inhibition of UCA1 ncRNA promotes bone formation via activation of the BMP-2/(Smad1/5/8) pathway in osteoblasts (Zhang et al., 2019). As a result, microRNAs and long ncRNAs remain among the most studied epigenetic factors involved in the pathogenesis of primary OP but require further systematisation.

## Epigenetic regulation of adipogenesis and osteoblastogenesis

The mechanisms of the relationship between bone and adipose cell formation are complex and remain an area of active research. Works performed on the cell cultures of osteoblasts and MSCs convincingly show an inverse relationship between differentiation of bone marrow MSCs into adipocytes or osteoblasts. An imbalance between adipogenesis and osteogenesis has been proposed as a mechanism for the development of OP, but obesity itself is not always a predictor of an increased risk of osteoporosis.

Post-translational modifications of histones play a key role in this system. Among them, histone methylation is crucial, in particular in chromatin reorganisation. In particular, lysine methylation in H4K20, H3K27 and H3K9 is associated with decreased levels of transcription, whereas methylation of H3K79, H3K36 and H3K4, with active gene transcription (Huang et al., 2015).

However, concerning osteogenic inducers, the homeobox gene HOXA10, which contributes to osteogenic clone determination by enriching the trimethylation of the 4th lysine residue in histone, activating RUNX2, alkaline phosphatase and osteocalcin, thus stimulating bone cell maturation, is important (Hassan et al., 2007). It is known that the combination of methylation and demethylation can function as an epigenetic switch of osteogenesis to adipogenesis based on EZH2 activity, catalysing the trimethylation of histone H3 on lysine 27 key regulatory genes (such as RUNX2). Simultaneously, removal of this tag by lysindemethylase 6A inhibits adipogenesis and induces osteoblastogenesis. Proteins that can be targeted by EZH2 and are involved in MSC switching are HDAC9c and HDAC (Chen et al., 2011). A direct correlation was realised between increased levels of EZH2 and decreased levels of HDAC9c gene expression (Chen et al., 2016). The methyltransferase activity of EZH2 is reduced by phosphorylation and is associated with osteogenic induction (Wei et al., 2011)

It is acknowledged that during osteocyte aging there is an accumulation of adipose tissue in the bone marrow and, at the same time, the number of mesenchymal stem cells increases in the intercellular phase. From this perspective, it is interesting that overexpression of miR-1292 accelerates the senescence of human adipose-derived stem cells and inhibits bone formation via the Wnt/β-catenin signalling pathway, while miR-10b inhibits adipose stem cell differentiation via the TGF-β pathway (Xu et al., 2020).

Several bone-associated cells, including multipotent bone mesenchymal stem cells, osteoblasts that form bone tissue and osteoclasts that break it down, are in a symbiotic relationship throughout life. A growing body of evidence suggests that epigenetic cell modifications induced by aging contribute to impaired bone remodelling and lead to osteoporosis. A variety of epigenetic mechanisms are involved, including DNA/RNA modifications, histone modifications, microRNAs (miRNAs) and long non-coding RNAs (dnRNAs), and chromatin remodelling (Yu et al., 2022). Thus, epigenetic mechanisms can switch the direction of mesenchymal stem cell differentiation between osteoblastogenesis and adipogenesis.

## Results of full-genome studies of methylome

Molecular genetic determinants of endophenotypes of osteoporosis, such as fracture risk and BMD levels, can be converged through the whole epigenome. In the research they conducted, J.A. Morris et al. (2017), leveraging the technological capabilities of Infinium HumanMethylation450, performed a whole-genome methylome analysis, measuring site-specific DNA methylation in 5515 individuals of European descent. Following a meta-analysis of their results, they were able to identify the CpG site cg23196985, which was significantly associated with low MPCT adjusted for multiple comparisons without regard to gender ( pBH = 1.30 × 10–2) and in females (pBH = 3.41 × 10–5).

The CpG cg23196985 site is localized to the 5′-translated region of the hepatic carboxylase 1 gene CES1, which is expressed in the liver and peripheral blood (Morris et al., 2017). J.G. Zhang et al. (2015), performing transcriptome analysis based on Affymetrix GeneChip Human Exon 1.0 ST Array and microRNA analysis on Capitalbio Cor. microarrays, as well as methylome sequencing in patients with low MPCT hip and controls, identified the most enriched functional molecular pathways associated with OP or MPCT variability: a network of 12 interacting genes and 11 microRNAs. Among the genes are AKT1, STAT5A, PIK3R5, etc., while among the microRNAs, miR-141 and miR-675 – their levels correlate with the expression of these genes and global DNA methylation status (Zhang et al., 2015).

D. Cheishvili et al. (2018) performed a full-epigenomic analysis in women without OP and in women with early postmenopausal OP. Genes in which CpG sites with significant levels of differential methylation were identified were ZNF267, ABLIM2, RHOJ, CDKL5, PDCD1, ABRA and HOJ (Cheishvili et al., 2018). At the human femur level, DNA methylation profile studies using pyrosequencing and qRT-PCR-based gene expression studies proved that DNA methylation status was inversely correlated with the expression of the iNOS and COL9A1 genes, but not catabolic genes including MMP13 and IL1B. Significant demethylation of the osteocalcin gene promoter was also shown between the embryonic and adult stages of development, demonstrating the importance of DNA methylation at the tissue level (Curtis et al., 2022). It is anticipated that the results of these studies will confirm the whole-epigenome approach as being sufficiently robust to allow large-scale studies of women at risk of developing OP.

## Conclusion

Despite the great advances and the wide range of work performed in the study of the epigenetics of primary osteoporosis, understanding remains fragmentary. The research describes key epigenetic regulators of bone remodelling, but it is difficult to build a coherent picture of the pathogenesis of osteoporosis from these data, common to all key pathogenetic processes in bone tissue.

It is recognised that the key epigenetic changes in osteoporosis are converging on the regulation of MSCs differentiation and that the multi-step system of activity regulation of the transcription factor RUNX2, sclerostin, DKK1 protein, RANKL-RANK-OPG system and factors involved in the regulation of WNT signalling is of great importance. A separate issue is the systematisation and creation of a unified genetic network of a vast number of regulatory microRNAs that affect key signalling pathways and the transcription factors associated with osteoporosis. However, these microRNAs influence the ultimate risk of osteoporosis indirectly and it remains to be understood how they can be systematised for relevance and functional involvement in pathogenesis due to their dynamism and different levels depending on tissue specificity.

Approaches necessary to implement early diagnosis or targeted therapies for osteoporosis still need to be developed. The task is complicated by experimental data from methylome studies, in which genes other than critical regulatory factors, such as RUNX2, sclerostin or DKK1, were observed to be key and most important. The data presented in this review show that epigenetic modifications can have a strong influence on the determination, differentiation and activity of mesenchymal stem cells and, therefore, may contribute to the pathophysiology of age-related bone mass loss. The results update further basic research on osteoporosis and, with the available data, broaden the horizons for a more insightful and detailed approach to new epigenetic studies of this disease and the prospects for new and effective personalised therapies

## Conflict of interest

The authors declare no conflict of interest.
